# Optimizing remote and rural prehospital resources using air transport of thrombectomy candidates

**DOI:** 10.1186/s13049-024-01203-3

**Published:** 2024-04-16

**Authors:** Pauli Vuorinen, Piritta Setälä, Sanna Hoppu

**Affiliations:** 1 Faculty of Medicine and Health Technology, University of Tampere, FI-33521 Tampere, PO Box 2000, Finland; 2 Emergency Medical Services, Centre for Prehospital Emergency Care, Wellbeing Services County of Pirkanmaa, Tampere, Finland

**Keywords:** Ambulance preparedness, Large vessel occlusion, Stroke, Helicopter emergency medical services

## Abstract

**Background:**

In Finland, the yearly number of mechanical thrombectomies for acute stroke is increasing and more patients are transported over 100 km to the comprehensive stroke centre (CSC) for definitive care. This leaves the rural townships without immediate emergency medical services (EMS) for hours. In this study we compare the EMS’ estimated return times to own station after the handover of a thrombectomy candidate between two transport methods: (1) using ground transportation with an ambulance to the CSC or (2) using a hydrid strategy starting the transportation with an ambulance and continuing by air with a helicopter emergency medical services unit (HEMS).

**Methods:**

We reviewed retrospectively all thrombectomy candidates’ transportations from the hospital district of South Ostrobothnia to definitive care at the nearest CSC, Tampere University Hospital from June 2020 to October 2022. The dispatch protocol stated that a thrombectomy candidate’s transport begins immediately with an ambulance and if the local HEMS unit is available the patient is handed over to them at a rendezvous. If not, the patient is transported to the CSC by ground. Transport times and locations of the patient handovers were reviewed from the CSC’s EMS database and the driving time back to ambulance station was estimated using Google maps. The HEMS unit’s pilot’s log was reviewed to assess their mission engagement time.

**Results:**

The median distance from the CSC to the ambulances’ stations was 188 km (IQR 149–204 km) and from the rendezvous with the HEMS unit 70 km (IQR 51–91 km, *p* < 0.001). The estimated median driving time back to station after the patient handover at the CSC was 145 min (IQR 117–153 min) compared to the patient handover to the HEMS unit 53 min (IQR 38–68 min, *p* < 0.001). The HEMS unit was occupied in thrombectomy candidate’s transport mission for a median of 136 min (IQR 127–148 min).

**Conclusion:**

A hybrid strategy to transport thrombectomy candidates with an ambulance and a helicopter reallocates the EMS resources markedly faster back to their own district.

## Introduction

Emergency calls and emergency medical services (EMS) utilization are increasing in western societies [1–3]. At the same time, the EMS has evolved from being only a transport service to out-of-hospital health care unit performing lifesaving interventions and health care guidance [4].

Some time-critical interventions still exist that require urgent transport preferably straight to definitive care. These interventions are typically centralized in metropolitan healthcare institutions. Mechanical thrombectomy of a large vessel occlusion (LVO) performed at a comprehensive stroke centre (CSC) is one of them [5]. LVO’s yearly incidence is estimated around 24 per 100 000 [6], however, the possibilities for treatment considering time limits and targeted arteries are evolving [7–9]. Optimal strategy for patient transport has been under debate for some time. Should a thrombectomy candidate be first transported to the nearest primary stroke centre (PSC) for diagnostics and possible thrombolysis or directly to the CSC? Irrespective of the chosen EMS transport strategy - either stopping at the PSC and confirming the LVO diagnosis or bypassing the PSC and transporting the patient directly for definitive care at the CSC [10] - the scarce ambulance resources in rural locations will lead to a situation where ambulances leave their own districts possibly for hours when transporting patients to the CSC. This inevitably enhances the risk for longer ambulance response times.

Various studies report time delays to reach mechanical thrombectomy when patients arrive from distant locations with different transport strategies [11–14]. However, no studies report how much time these units use on their way back to their district or when they will be available for the next patient.

We hypothesized that rural ambulance availability increases if a helicopter emergency medical services (HEMS) unit is utilized to expedite the transportation of a thrombectomy candidate to definitive care. To examine this, we investigated how the ambulances were occupied in the thrombectomy candidates’ transportations in two transport methods and their estimated times of arrival back to their own stations after the patient handover either at the CSC or at the rendezvous with a HEMS unit continuing the transport to the CSC. Also, we report the time the HEMS unit was engaged in the missions and its parallel dispatches.

## Subjects and methods

This is a secondary analysis of our data from 72 EMS missions for thrombectomy candidates transported to our CSC, Tampere University Hospital, and described in our earlier publication, which also elucidates the setting and the protocol [14]. Briefly, in June 2020 we launched a new protocol where we dispatched the Tampere University Hospital’s HEMS unit, FinnHEMS30, to expedite the thrombectomy candidates’ transportations from the hospital district of South Ostrobothnia for definitive care in the CSC. During this study FinnHEMS30 was the only HEMS unit capable of reaching South Ostrobothnia hospital district [15]. Seinäjoki central hospital is the only primary stroke centre (PSC) in this region performing circa 50 thrombolyses in a year for acute stroke. The distance between these two hospitals is around 150 km by air and 175 km by road. The EMS in South Ostrobothnia are dispatched to 36,000 missions per year and around 1500 of these are defined as suspected stroke. Approximately 700 patients are transported to the PSC as candidates for recanalization. During the study, a prehospital physician manned rapid response vehicle operated in South Ostrobothnia. They attended, for example, EMS missions concerning major trauma and out-of-hospital cardiac arrest but not stroke. The paramedics in a predefined region consulted the on-call neurologist at the Tampere university hospital when the patient had symptoms highly indicative of an LVO. Suspected stroke patients were screened with the Finnish Prehospital Stroke Scale and the directly to CSC -strategy was chosen when applicable [16]. More specifically, LVO was suspected if the patient presented with hemiparesis and conjugate eye deviation away from the side of the hemiparesis. The paramedics were instructed to commence the thrombectomy candidate’s transport to the CSC as soon as possible. They also notified the national emergency response centre agency to dispatch the HEMS unit to continue the transport after a rendezvous. We called this method the hybrid transport. When the HEMS unit was occupied in another mission, or the weather conditions were unsuitable for air transport, the patients’ transportation to the CSC continued with ground EMS. This dispatch protocol was terminated in October 2022 when a new HEMS unit, FinnHEMS40, commenced operating from Seinäjoki airport.

We identified the EMS route and the rendezvous with the HEMS unit using a web reporting portal (Codea Ltd, Porvoo, Finland). We used Google maps (Google LLC, Mountain View, Cal, USA) to estimate the shortest driving time back to the ambulance’s station from the point where the air transport began or from the CSC if the HEMS unit was not able to transport the patient. To determine the HEMS unit’s availability, we reviewed the flight operator’s log to define the time when the HEMS unit was ready for the next operation. The primary outcome was the estimated driving time of the ambulance back to their own station. Secondarily, we report the total time the ambulance and the HEMS unit were engaged in the mission, i.e. from dispatch until arrival at their respective stations depending on whether the PSC was bypassed or not.

Collected data was analysed using SPSS statistical software (IBM Corp., Armonk, NY, USA) version 26. Mann-Whitney U test was used for data comparison. The tests were two-sided, and *p* < 0.05 was considered significant.

The institutional review board of Tampere university hospital approved the original study design (IRB number R20082R). This study contains no patient data and hence patient consent was not applicable.

## Results

**Fig. 1:**
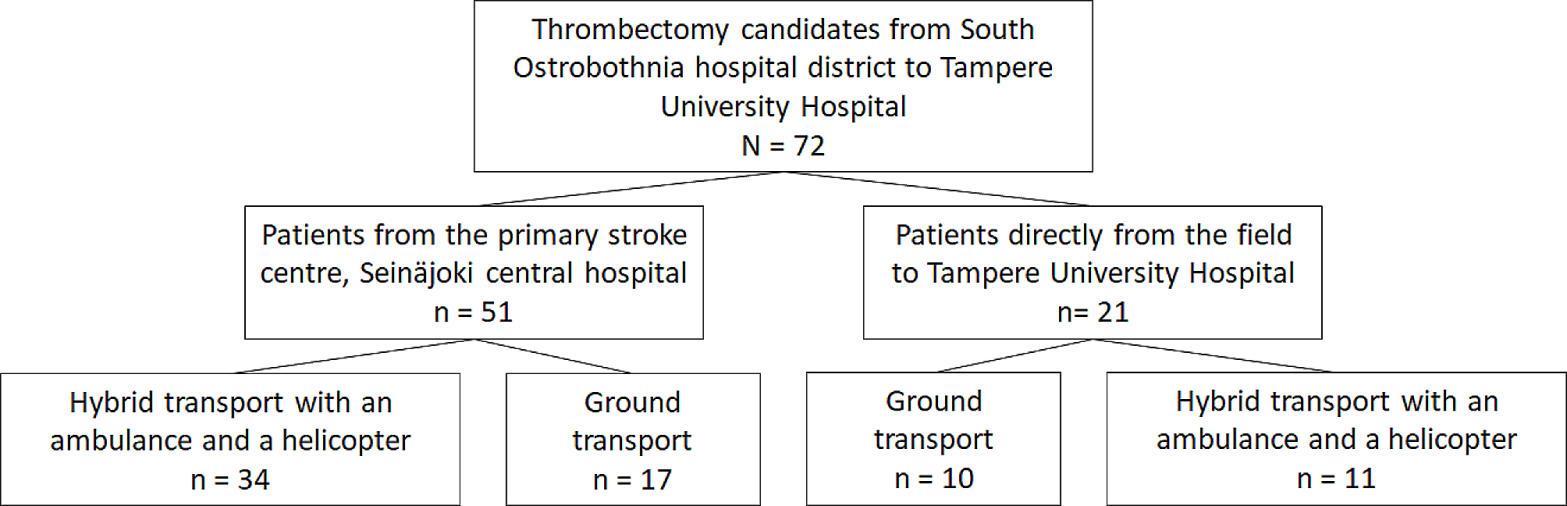
Flow chart of the study

Of the 72 thrombectomy candidate transports, 45 (63%) were completed using HEMS. Of these 45 hybrid transports 34 began with an ambulance from the PSC and in 11 air lifts the directly to CSC -strategy was chosen. Seventeen patients were transported from the PSC to the CSC with an ambulance only and in ten cases the ambulance bypassed the PSC driving all the way to the CSC. Figure 1 is the flow chart of the patients in each transport strategy.

The median distance from the CSC to the ambulances’ stations was 188 km (IQR 149–204 km) km and the median distance from the rendezvous location with the HEMS unit was 70 km (IQR 51–94 km, *p* < 0.001). The median EMS driving time back to their own station after the patient handover at the CSC was 145 min (IQR 117–153 min) compared to 53 min (IQR 37–68 min, *p* < 0.001) after the patient handover to the HEMS unit. The ambulances total engagement times in the missions were also considerably shorter when the hybrid transport was utilized. Table 1 shows the key results of the study.

**Table 1:** Key results of the study

	Hybrid (*n* = 45)		Ground (*n* = 27)		
	median	IQR	median	IQR	p
Distance back to station after patient handover (km)	70	51–94	188	149–204	< 0.001
Estimated driving time back to station after patient handover (h: min)	0:53	0:37–1:08	2:25	1:57–2:33	< 0.001
Time the ambulance was occupied in the mission when transporting the patient via PSC (h: min)	3:32	2:42–4:23	6:24	5:23–6:44	< 0.001
Time the ambulance was occupied in the mission when transporting the patient straight to the CSC (h: min)	2:12	1:48–2:50	4:14	3:57–4:47	< 0.001
Time the HEMS unit was occupied in the mission (h: min)	2:16	2:07–2:28			

CSC: Comprehensive stroke centre, EMS: emergency medical services, PSC: Primary stroke centre

The HEMS unit was occupied in thrombectomy candidate’s transport for a median of 136 min (IQR 127–148 min). The HEMS unit was notified of a parallel mission 17 times during 14 air transports. Four of these missed EMS missions ended up in non-conveyance, six in low-urgency transport and four in high-urgency transport. Three times the parallel dispatch concerned out-of-hospital cardiac arrest and the resuscitation attempt was terminated on the scene due futility.

**Fig. 2:**
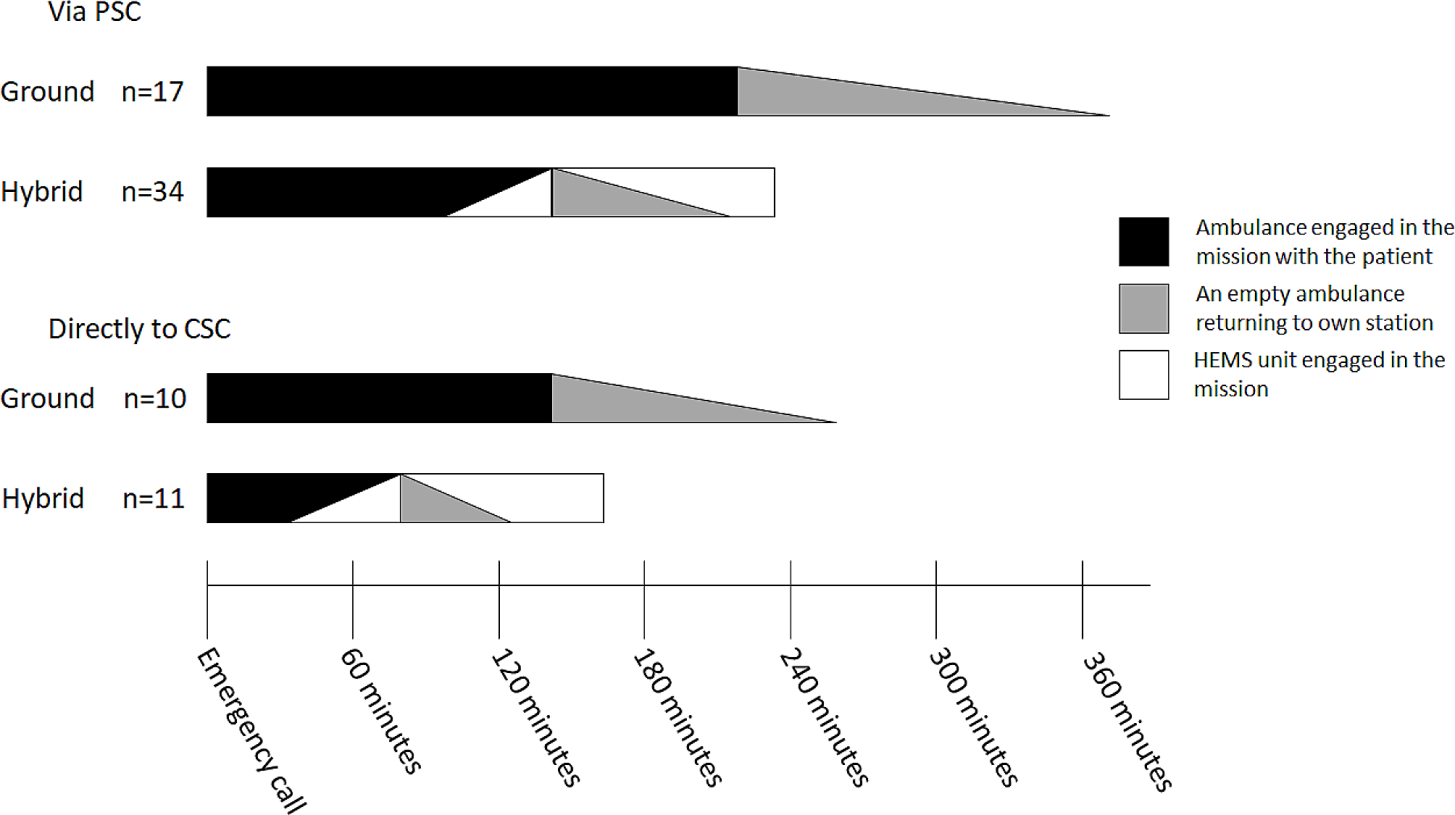
Median times of the (H)EMS crews were engaged in thrombectomy candidates’ transports. In the via PSC -strategy the patient is first diagnosed at the primary stroke centre. In the directly to CSC -strategy the ambulance bypasses the nearest stroke centre and transports the patient directly to the comprehensive stroke centre. “Ground” refers to ambulance transport, “Hybrid” refers to expedited transport with an ambulance and a helicopter. HEMS: Helicopter Emergency Medical Services

Figure 2 shows the median times of the ambulances and the HEMS unit engaged in the transport and how they intertwine with different transport strategies.

## Discussion

The present study describes the effect of different transport methods to the time the ambulance is back in own district after conveying a thrombectomy candidate to definitive care. We estimate that our hybrid method of transporting a thrombectomy candidate with an ambulance and a HEMS unit reduces the time without a local ground EMS unit in rural environment. To our knowledge, this is the first study on the subject.

In prehospital care, mathematical models have been introduced to deploy ambulance repositioning. These models might be feasible in environments with high population density, short distance to the receiving hospital and a high number of EMS dispatches [17]. The case is completely different when patients are transported long distances to the CSC for definitive care from rural communities where EMS units are sparsely distributed.

Allocating a specialized ambulance for the interfacility transfer prevents the regional decrease in ambulance preparedness. When viewing the system’s performance this might be the choice [18] but not feasible for time-critical thrombectomy candidates. We concur with the previously suggested protocol aiming at decreasing the door-in-door-out time: the paramedics conveying a thrombectomy candidate to the primary stroke centre should stay with the patient until there is a final decision whether the patient will continue to the CSC or is admitted to the stroke unit at the local institution [19]. We showed very competitive door-in-door-out times in our previous study using this strategy [14, 20, 21]. 

It is inevitable that the HEMS crew must prioritize missions. Our HEMS unit was occupied transporting a thrombectomy candidate 17 times when they were dispatched for another callout. At least 10 of these dispatches proved to be overtriaged from the HEMS unit’s perspective. This percentage is common in Nordic HEMS [15, 22–24]. Optimal HEMS dispatch balances between immediate dispatch and precision [25]. Behrndtz et al. [26] suggested in their theoretical model that two helicopters per HEMS base could be the solution. With one helicopter per base, they approximated the cost of one patient benefitting from the air transport to be around 150 000 euros. On the other hand, setting up a new advanced life support level ambulance to the PSC area to increase the ambulance preparedness when one of the local ambulances is on the way to the CSC does not come without expenses. Hubert et al. [12] present a highly innovative protocol in their report about “flying intervention teams”. Investing in an extra helicopter with two pilots and in an on-call intervention team decreased the time from onset of symptoms to recanalization and left the dedicated HEMS unit vacant for out-of-hospital emergencies and the local ambulance was able to return to own station. Regrettably, HEMS unit utilization has not been distinctively proven to be beneficial for the thrombectomy candidate [11, 12, 14, 27–29]. Expediting the ambulance’s return to station could be valuable for the next emergency but we fear it is impossible to demonstrate this in practice. Nevertheless, a prospective study gathering the data when the ambulances transporting LVO patients to the CSC actually depart from the CSC, when they return to their own station, and when they assign for the next dispatch could be easily completed. The HEMS unit’s role in the care of thrombectomy candidates who are later diagnosed with an intracerebral haemorrhage is unsolved. These patients are shown to have a dismal prognosis and they could benefit from prehospital airway interventions [30].

## Strengths and limitations

This is a single centre study in which the driving time between the primary EMS mission and the CSC is more than 90 min. One cannot extrapolate these results into metropolitan districts where ambulance’s reallocation after a thrombectomy candidate’s might not be as big of a problem.

We used the Codea web reporting portal to track the ambulances’ routes to the CSC and the rendezvous with the HEMS unit. However, the tracking ends when the paramedics register the mission finished in their computer. Hence, we were not able to determine their actual return time to station. This leaves us with mere estimates of the return which is the greatest limitation of this study. Additionally, the ambulance crew may need to supplement their equipment and not being able to take part in the next dispatch immediately after entering own hospital district. Nevertheless, we present results closer to real world experience than mathematical modelling of predicted future missions.

## Conclusion

Hybrid method of transporting thrombectomy candidates to definitive care is a feasible way of increasing the availability of the ambulance service in rural locations.

## Data Availability

The datasets in the study are available from the corresponding author on reasonable request.

## Abbreviations

CSC: Comprehensive Stroke Centre.
EMS: Emergency Medical Services.
HEMS: Helicopter Emergency Medical Services.
LVO: Large Vessel Occlusion.
PSC: Primary Stroke Centre.
